# Pancreatic Tail Schwannoma in a 44-Year-Old Male: A Case Report and Literature Review

**DOI:** 10.1155/2013/416713

**Published:** 2013-11-25

**Authors:** Ahmed Abu-Zaid, Ayman Azzam, Hussam Abou Al-Shaar, Abdullah M. Alshammari, Tarek Amin, Shamayel Mohammed

**Affiliations:** ^1^College of Medicine, Alfaisal University, P.O. Box 50927, Riyadh 11533, Saudi Arabia; ^2^Department of Surgical Oncology, King Faisal Specialist Hospital and Research Center (KFSH&RC), P.O. Box 3354, Riyadh 11211, Saudi Arabia; ^3^Department of General Surgery, Faculty of Medicine, Alexandria University, Alexandria 21526, Egypt; ^4^Department of Pathology and Laboratory Medicine, King Faisal Specialist Hospital and Research Center (KFSH&RC), P.O. Box 3354, Riyadh 11211, Saudi Arabia

## Abstract

Pancreatic schwannomas are exceedingly uncommon neoplasms. According to a recent study in 2012, less than 50 cases of pancreatic schwannoma have been described in the English literature over the past thirty years. The vast majority of pancreatic schwannomas take place in the head and body of pancreas, respectively. Herein, we report the case of pancreatic tail ancient schwannoma in a 44-year-old man who presented with a 4-month history of epigastric pain. On physical examination, epigastric region was moderately tender to palpation without evidence of a palpable mass. All laboratory tests were normal. Contrast-enhanced computed tomography (CT) scan showed a 9.2 × 9.5 × 11.5 cm, huge, and well-defined left suprarenal mass arising either from adrenal gland, pancreas, or retroperitoneum. The mass demonstrated mild heterogeneous enhancement with central cystic/necrotic area. No evidence of distant metastasis was identified. At laparoscopy, the mass was noticed to originate from pancreatic tail. Patient underwent surgical resection of pancreatic tail. Microscopic and immunohistochemical examination of the pancreatic tail specimen showed ancient schwannoma. Patient received no adjuvant therapy. At a postoperative 6-month followup, patient was completely asymptomatic and CT scan imaging showed no evidence of tumor recurrence. Moreover, a literature review on pancreatic schwannomas is presented.

## 1. Introduction

Pancreatic schwannomas are exceedingly uncommon neoplasms. According to a recent study in 2012, less than 50 cases of pancreatic schwannoma have been described in the English literature over the past thirty years [1]. The vast majority of pancreatic schwannoma cases take place in the head (40%) and body (20%) of pancreas, respectively [1–3]. Herein, we report the case of pancreatic tail ancient schwannoma in a 44-year-old man who presented with a 4-month history of epigastric pain. Moreover, a literature review on pancreatic schwannomas is presented.

## 2. Case Report

A 44-year-old man presented with a 4-month history of vague epigastric pain. The pain was gradually increasing in severity and associated with nausea and vomiting. Patient denied any history of physical trauma, gastroesophageal reflux disease, gastritis, peptic ulcer disease, pancreatitis, or hepatobiliary disease. Moreover, patient denied any history of constitutional symptoms such as fever, night sweating, or weight loss. Past medical, surgical, and family history were unremarkable.

On physical examination, patient was vitally stable. Abdomen was soft, lax, and nondistended. However, the epigastric region was moderately tender to palpation without evidence of a palpable mass.

All laboratory tests including complete blood count, hepatic, coagulation renal and bone profiles, amylase, lipase, lactate dehydrogenase (LDH), carcinoembryonic antigen (CEA), and cancer antigen 19-9 (CA 19-9) were within normal values.

**Figure 1:**
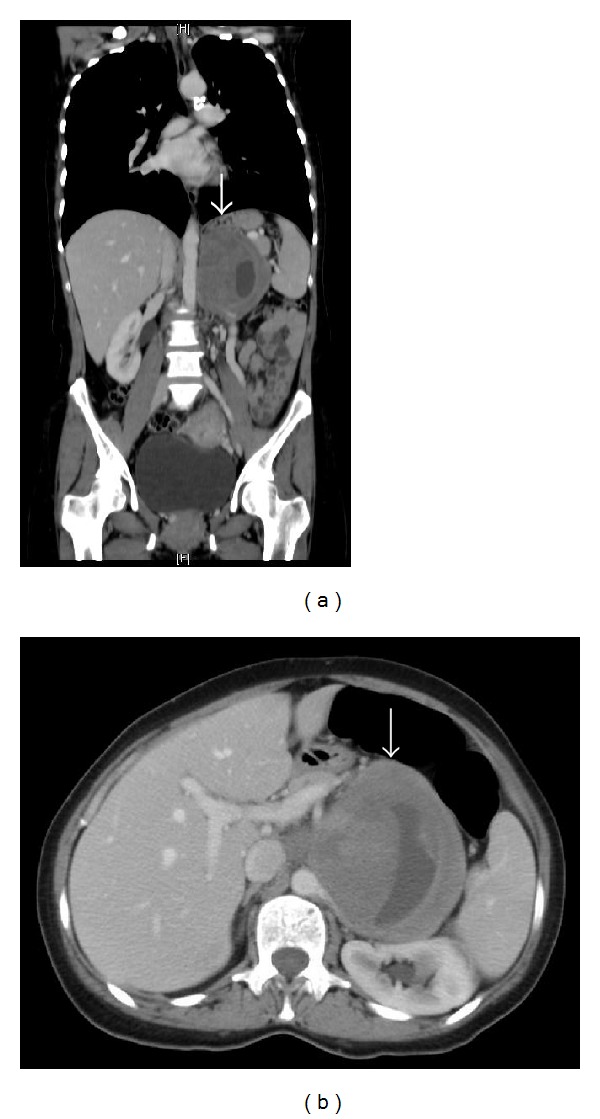
Coronal (a) and transverse (b) contrast-enhanced computed tomography (CT) scan showed a 9.2 × 9.5 × 11.5 cm, huge, and well-defined left suprarenal mass (arrow) arising either from adrenal gland, pancreas, or retroperitoneum. The mass demonstrated mild heterogeneous enhancement with central cystic/necrotic area. No evidence of lymphadenopathy or regional/distant metastasis was identified.

Contrast-enhanced computed tomography (CT) scan showed a 9.2 × 9.5 × 11.5 cm, huge, and well-defined left suprarenal mass arising either from adrenal gland, pancreas, or retroperitoneum. The mass demonstrated mild heterogeneous enhancement with central cystic/necrotic area. No evidence of lymphadenopathy or regional/distant metastasis was identified (Figures 1(a) and 1(b)).

At laparoscopy, the mass was noticed to originate from pancreatic tail. Patient underwent surgical resection of pancreatic tail, splenectomy, and celiac lymph node dissections. Macroscopic and microscopic examination of spleen and celiac lymph node dissections revealed unremarkable pathological findings and were negative for malignant components.

**Figure 2:**
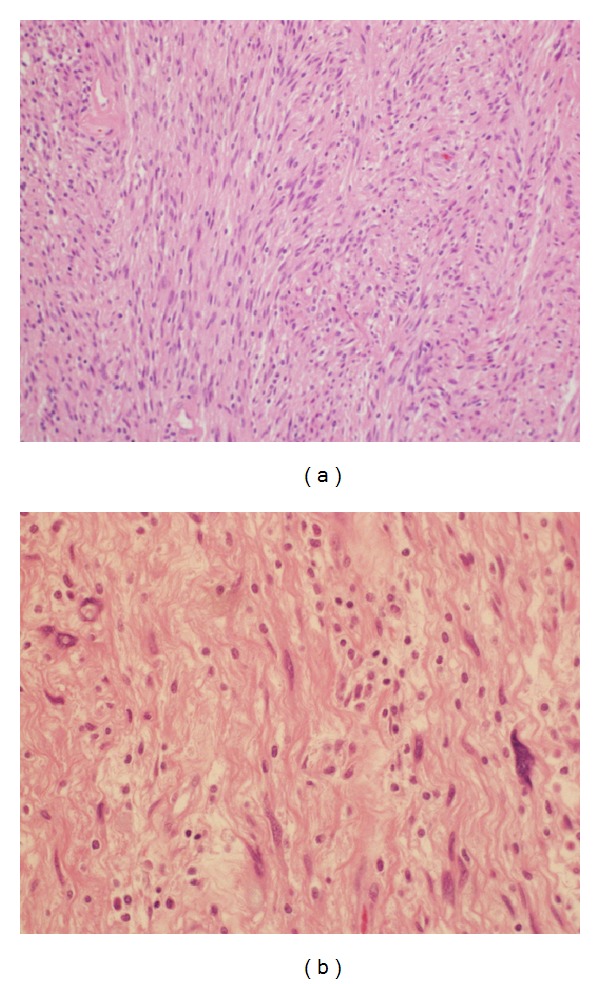
Microscopic examination of the pancreatic tail tumor. (a) Tumor was composed of monomorphic spindle-shaped Schwann cells with poorly defined eosinophilic cytoplasm and pointed basophilic nuclei set in a collagenous stroma. Focal nuclear palisading is noted (H&E stain, magnification power: 10x). (b) Areas characteristic for Antoni B composed of Schwann cells with inconspicuous cytoplasm and nuclei suspended in myxoid matrix are identified. Scattered degenerated nuclei in a hyalinized stroma suggestive of ancient schwannoma were present. No mitosis or necrosis was noted (H&E stain, magnification power: 40x).

**Figure 3:**
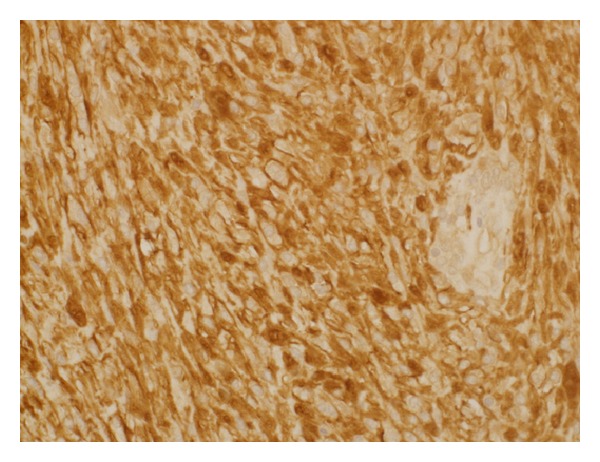
Immunohistochemical examination of the pancreatic tail tumor showed diffuse and strong positivity to S100 protein (magnification power: 40x).

Macroscopically, the distal pancreatic mass measured 7.1 × 6.2 cm. A cut section showed solid and yellow mass with a central cystic area. Microscopically, the distal pancreatic mass was composed of monomorphic spindle-shaped Schwann cells with poorly defined eosinophilic cytoplasm and pointed basophilic nuclei set in a collagenous stroma. Focal nuclear palisading was noted (Figure 2(a)). Areas characteristic of Antoni B composed of Schwann cells with inconspicuous cytoplasm and nuclei suspended in myxoid matrix were identified. Scattered degenerated nuclei in a hyalinized stroma suggestive of ancient schwannoma were present. No mitosis or necrosis was noted (Figure 2(b)). Immunohistochemically, tumor cells stained diffusely and strongly positive for S100 protein (Figure 3). Conversely, tumor cells stained negative for CD34, CD117, CK, and ALK-1. A final diagnosis of primary ancient schwannoma of pancreatic tail was established.

Patient had an uneventful postoperative course following surgery. Postoperatively, patient received no adjuvant therapy. At a postoperative 6-month followup, patient was completely asymptomatic and CT scan imaging showed no evidence of tumor recurrence.

## 3. Discussion

Pancreatic schwannomas are exceedingly uncommon neoplasms. According to a recent study in 2012, less than 50 cases of pancreatic schwannoma have been described in the English literature over the past thirty years [1]. Although the vast majority of these neoplasms are benign [4–6], malignant neoplasms should not be excluded. Five cases of malignant pancreatic schwannomas have been documented in the English literature [1, 2, 4–6].

Pancreatic schwannomas arise from specialized myelin-producing cells (Schwann cells) located on the nerve sheath of the peripheral epineurium of either the sympathetic or parasympathetic autonomic fibers. These autonomic fibers travel the pancreas via the vagus nerve [6–9]. Pancreatic schwannomas commonly affect adults (range: 20–87 years; mean age of diagnosis is roughly 56 years) [1]. In addition, males and females are relatively equally affected [1, 6–8, 10, 11].

Pancreatic schwannomas vary in size and location [2, 3, 12]. They range from 1 to 20 cm in diameter (mean neoplasm size is approximately 6 cm), with the head of pancreas being involved in the vast majority of cases (40%), followed by its body (20%) [1–3]. However, other pancreatic areas are also prone to develop these neoplasms [2, 13]. An association between the tumor size, malignant potential, and cystic formation has been described in the literature; that is, the larger the tumor, the more likely to be malignant and undergo cystic degeneration, whereas the smaller the tumor, the more likely to be benign and solid (or mixed) [1].

Pancreatic schwannomas generally grow slowly accounting for their benign potential [7, 10]. Studies have demonstrated that more than two-thirds of pancreatic schwannomas undergo degenerative changes including cyst formation, hemorrhage, calcification, xanthomatous infiltration, and hyalinization, which frequently can be confused with closely related pancreatic cystic lesions on radiographic imaging [1, 4–6, 10–12, 14, 15]. Therefore, cystic schwannomas should be included in the differential diagnosis of pancreatic cystic lesions, which also encompass nonfunctioning endocrine neoplasms, pancreatic pseudocysts, mucinous and serous cystic neoplasms, solid and pseudopapillary neoplasms, cystadenomas, cystadenocarcinomas, and lymphangiomas [1, 3, 14–17].

A recently published study in 2012 has shown that 70% of the reported pancreatic schwannoma patients were symptomatic [1]. Symptoms were often vague and nonspecific. The most common presenting symptom was nonspecific abdominal pain (60%). Weight loss, back pain, nausea and/or vomiting, abdominal mass, anemia, melena, jaundice, and gastrointestinal bleeding have also been reported in a descending order of frequency [1, 5, 18, 19]. However, about 30% of the diagnosed pancreatic schwannoma patients were asymptomatic [1].

Laboratory investigations are not usually useful in the diagnosis of pancreatic schwannomas [3, 8]. Hematological, hepatic, and renal profiles; serum amylase, and tumor markers including cancer antigen 19-9 (CA 19-9) and carcinoembryonic antigen (CEA) usually lie within normal values [2, 3, 8].

Diagnosing pancreatic schwannoma is challenging as it is frequently confused with other cystic lesions [3, 10, 13]. Diagnosing these tumors prior to operation is tremendously difficult [3, 8, 13]. Preoperative definitive diagnosis is not possible even with recent imaging techniques and laboratory tests. Definitive diagnosis can only be made through histopathological examination and immunohistochemical staining of the neoplasm, which cannot be established without a biopsy of the tumor [10, 20, 21].

A variety of diagnostic imaging modalities can be utilized to identify pancreatic schwannomas. Computed tomography (CT) scan can frequently demonstrate the cystic and/or solid components of tumor [3, 8, 9, 13, 22, 23]. Well-defined hypodense lesions with encapsulation and/or cystic degeneration are common findings on the CT scan [13, 20, 22, 23]. The Antoni B components of tumor (which commonly undergo degenerative changes) can be appreciated as low density and/or cystic degenerative areas on the CT scan [13, 22, 23]. Moreover, after the administration of a contrast agent, Antoni A and Antoni B components of the tumor can be well distinguished from each other on the CT scan. Antoni A areas are usually enhancing lesions (do uptake the contrast) whereas Antoni B areas are frequently nonenhancing lesions (do not uptake the contrast) [13, 22, 23]. Therefore, Antoni A areas are often more vascular than Antoni B areas [13, 22, 23].

Magnetic resonance imaging (MRI) can usually outline the degree of vascular involvement of the tumor, which may be greatly helpful in differentiating the potential biological behavior of the lesion in terms of being benign or malignant [9, 20, 22, 24]. Pancreatic schwannomas typically appear hypointense on T1-attenuated images and hyperintense on T2-attenuated images [9, 14, 20, 22, 24, 25].

Ultrasound (US) can also be used in the diagnosis of pancreatic schwannomas. Tumors that are entirely cystic or have some cystic components appear hypoechoic on US. In addition, the topographies of the solid component of the tumor can be appreciated on US more clearly than CT or MRI [13]. However, all of these radiological findings are not specific and can be noticed with other pancreatic cystic lesions [13].

The effectiveness of ultrasonography-guided (US-guided) fine needle aspiration (FNA) in diagnosing pancreatic schwannomas remains a point of dispute. Owing to the insufficient specimen collection and/or defects in the collection technique, a study has shown that only one of eight histologically proven schwannomas can be diagnosed correctly using US-guided FNA, which imposes a huge limitation regarding the efficiency of this modality in diagnosing pancreatic schwannomas [2, 3, 26, 27].

Macroscopically, pancreatic schwannomas commonly appear cystic [1, 3, 13]. However, solid and mixed tumors have also been reported [1, 13]. Typically, a well-demarcated, encapsulated, homogeneous, tan-yellow, round nodule with or without myxomatous and/or hemorrhagic areas is seen within the pancreatic parenchyma [1, 2, 13].

Microscopically, typical pancreatic schwannomas appear to contain two distinctive areas: Antoni A and Antoni B areas. Antoni A area is characterized by hypercellular region of closely packed long bipolar cells (spindle cells) arranged in palisading and interlacing fashions. Verocay bodies without mitotic figures can also be present. Conversely, Antoni B area is characterized by loose hypocellular region exhibiting degenerative changes such as cyst formation, hemorrhage, calcification, xanthomatous infiltration, and hyalinization [9, 28, 29]. Vascular thrombosis with consequent necrosis largely accounts for the changes that are commonly observed in Antoni B areas [29]. However, it must be noted that both Antoni A and Antoni B areas have been reported in widely variable proportions in the vast majority of pancreatic schwannoma cases [1, 13].

Immunohistochemically, pancreatic schwannomas diffusely and strongly stain positive for S100 protein. In addition, they occasionally stain positive for CD56 and vimentin. Conversely, spindle cells in pancreatic schwannomas stain negative for cytokeratin, CD117, desmin, CD34, AE1/AE3, alpha smooth muscle actin, and smooth muscle myosin [13, 21, 29].

Management of pancreatic schwannomas remains largely controversial. However, pancreatic schwannomas generally behave benignly and malignant transformation of these neoplasms is extremely unlikely [1–3, 21]. Therefore, if definitive histopathological diagnosis can be established before or during surgery, simple enucleation of these tumors is usually achievable, safe, and sufficient [1–3, 8, 21]. Nevertheless, if the tumor has proven to be malignant or definitive diagnosis cannot be established before or during surgery; then oncological surgical resection is required [2, 21]. Radical surgical resection is required and type of pancreatectomy is decided according to the involved region of pancreatic schwannoma [1, 3, 8, 13]. Both enucleation and radical surgical resections have revealed great therapeutic efficiency as demonstrated by the absence of documented mortality, severe morbidity, and tumor recurrence in all the pancreatic schwannoma cases managed by either modality [1–3].

Intraoperative frozen section should be carried out in all pancreatic schwannoma cases. Complete histopathological examination and immunohistochemical staining are crucial in order to obtain an accurate diagnosis and avoid the unnecessary radical resection for benign lesions [1].

The utilization of radiation therapy in the management of unresectable pancreatic schwannomas has not been established yet [5]. Nonetheless, neurogenic schwannomas have been managed with radiation therapy for decades. Radiation therapy for neurogenic schwannomas has proven to be effective in terms of slowing down their growth rates as well as shrinking their sizes considerably [30, 31]. With all these new innovations in the management of pancreatic schwannomas, surgical resection of tumors—whenever technically possible—along with continuous followups remains the standard of care in the management of pancreatic schwannomas [3].
